# Comparative analyses of angiosperm secretomes identify apoplastic pollen tube functions and novel secreted peptides

**DOI:** 10.1007/s00497-020-00399-5

**Published:** 2020-11-30

**Authors:** María Flores-Tornero, Lele Wang, David Potěšil, Said Hafidh, Frank Vogler, Zbyněk Zdráhal, David Honys, Stefanie Sprunck, Thomas Dresselhaus

**Affiliations:** 1 Cell Biology and Plant Biochemistry, University of Regensburg, Universitätsstraße 31, 93053 Regensburg, Germany; 2 Mendel Centre for Plant Genomics and Proteomics, Central European Institute of Technology, Masaryk University, Kamenice 5, 62500 Brno, Czech Republic; 3 Laboratory of Pollen Biology, Institute of Experimental Botany ASCR, Rozvojová 263, 165 02 Prague 6, Czech Republic

**Keywords:** Pollen tube, Proteomics, Secretome, Cell wall, CRP, Signaling, Amborella, Maize, Tobacco

## Abstract

**Key message:**

Analyses of secretomes of in vitro grown pollen tubes from Amborella, maize and tobacco identified many components of processes associated with the cell wall, signaling and metabolism as well as novel small secreted peptides.

**Abstract:**

Flowering plants (angiosperms) generate pollen grains that germinate on the stigma and produce tubes to transport their sperm cells cargo deep into the maternal reproductive tissues toward the ovules for a double fertilization process. During their journey, pollen tubes secrete many proteins (secreted proteome or secretome) required, for example, for communication with the maternal reproductive tissues, to build a solid own cell wall that withstands their high turgor pressure while softening simultaneously maternal cell wall tissue. The composition and species specificity or family specificity of the pollen tube secretome is poorly understood. Here, we provide a suitable method to obtain the pollen tube secretome from in vitro grown pollen tubes of the basal angiosperm *Amborella trichopoda* (Amborella) and the Poaceae model maize. The previously published secretome of tobacco pollen tubes was used as an example of eudicotyledonous plants in this comparative study. The secretome of the three species is each strongly different compared to the respective protein composition of pollen grains and tubes. In Amborella and maize, about 40% proteins are secreted by the conventional “classic” pathway and 30% by unconventional pathways. The latter pathway is expanded in tobacco. Proteins enriched in the secretome are especially involved in functions associated with the cell wall, cell surface, energy and lipid metabolism, proteolysis and redox processes. Expansins, pectin methylesterase inhibitors and RALFs are enriched in maize, while tobacco secretes many proteins involved, for example, in proteolysis and signaling. While the majority of proteins detected in the secretome occur also in pollen grains and pollen tubes, and correlate in the number of mapped peptides with relative gene expression levels, some novel secreted small proteins were identified. Moreover, the identification of secreted proteins containing pro-peptides indicates that these are processed in the apoplast. In conclusion, we provide a proteome resource from three distinct angiosperm clades that can be utilized among others to study the localization, abundance and processing of known secreted proteins and help to identify novel pollen tube secreted proteins for functional studies.

**Electronic supplementary material:**

The online version of this article (10.1007/s00497-020-00399-5) contains supplementary material, which is available to authorized users.

## Introduction

In flowering plants (angiosperms), sperm cells have lost their mobility and are transported as a passive cargo by pollen tubes deep into the maternal tissues of the ovary toward ovules (Zhang et al. 2017). Inside ovules, sperm cells are released for a double fertilization process to generate an embryo and endosperm, respectively (Dresselhaus et al. 2016). During their journey, pollen tubes communicate intensively with female reproductive tissues starting with papilla cells of the stigma during germination, transmitting tract cells during further growth, maternal cells of the ovule as well as cells of the female gametophyte (embryo sac) for guidance and attraction culminating in pollen tube burst (Dresselhaus and Franklin-Tong 2013; Johnson et al. 2019; Zhou and Dresselhaus 2019). Interaction with the synergid cells, secretory cells of the embryo sac, is especially critical as these regulate attraction during the last passage of the journey and termination during sperm cell perception (Maruyama and Higashiyama 2016).

During their journey, pollen tubes therefore secrete many different types of proteins and peptides (secretome) (Johnson et al. 2019; Qu et al. 2015). The secretome is generally considered as the sum of proteins secreted into the extracellular space of a plant cell or tissue at any given time and under certain conditions through various secretory mechanisms (Agrawal et al. 2010). Although it is difficult to identify secretome components in vivo, genetic and transcriptomic studies have uncovered that pollen tubes secrete various types of signaling ligands including chemocyanins and plantacyanins (Chae and Lord 2011) as well as small cysteine-rich proteins (CRPs) like LAT52 (Muschietti et al. 1994), rapid alkalinization factors (RALFs) (Ge et al. 2017; Mecchia et al. 2017) and lipid transfer proteins (LTPs) (Chae et al. 2010). The roles of some secreted peptides/proteins in pollen tube growth, guidance or cell wall integrity have been elucidated. CRPs appear especially important as they are overrepresented in pollen tubes compared with vegetative tissues (Bircheneder and Dresselhaus 2016; Huang et al. 2015). Additionally, many secreted proteins are involved in formation, modification and remodeling of the pollen tube cell wall, such as expansins, pectin methylesterases (PMEs) and their inhibitors (PMEIs), pectin lyases and glycoside hydrolases (Dehors et al. 2019; Mollet et al. 2013).

To reach the extracellular space, it was initially thought that most proteins secreted from pollen tubes contain a *N*-terminal signal peptide to be targeted and processed through the classical/conventional secretory route via the endoplasmic reticulum, Golgi and trans-Golgi network. However, recent studies have shown that about 50% secreted plant proteins lack known *N*-terminal signal peptides indicating alternative/unconventional secretion pathways involving, for example, the EXPO complex (Krause et al. 2013). Until now, only few studies support the presence of unconventional secretory pathways also for pollen tube secretions as shown for *Olea europaea* (olive tree) (Alché et al. 2004; Prado et al. 2014) and *Nicotiana tabacum* (tobacco) (Hafidh et al. 2016), respectively.

Despite these studies, our current knowledge about the exact composition and function of the protein/peptide components of pollen tube secretomes is far from being complete. In order to elucidate secretome components of pollen tubes from various plant species, we describe here an easy and reproducible protocol that can be widely applied to identify secreted proteins from in vitro grown pollen tubes. The basal angiosperm *Amborella trichopoda*, the monocotyledonous crop and grass model *Zea mays* (maize) and the eudicotyledonous plant *Nicotiana tabacum* (tobacco) have then been used as representatives of different angiosperm clades to compare the components of their respective secretomes, the prevalence of conventional versus unconventional secretory pathways and associated functions of secreted proteins. Finally, we show how this approach can be used to identify novel secreted small peptides and how the secretome can be exploited to elucidate, for example, processing of secreted proteins.

## Materials and methods

### Plant material and growth conditions

Male flowers of *Amborella trichopoda* (Amborella) were harvested at the Botanical Garden in Bonn (Germany). Plants were grown in a shaded place inside a greenhouse under controlled conditions of 16–18ºC, constant humidity of about 66% and 12-h photoperiods. Fully opened male flowers were gathered in 50-ml Falcon™ conical tubes (Thermo Fisher), placed without lid in a hermetically sealed plastic box containing a bed of silica gel and stored at 4 ºC until pollen isolation. Mature pollen grains from *Zea mays* (maize) were obtained from B73 inbred line. Seeds were germinated in a humid chamber and transferred 5 days later to individual 10-cm-diameter pots with a soil and substrate mixture (1:1, v/v). Maize seedlings were then transferred to 10-L pots in the greenhouse under controlled conditions of a constant air humidity of 60–65%, 14 h of light at 26 ºC and 10 h of darkness at 21 ºC. Supplemented light of 16,000 lx was provided to adjust day length duration. Plants were watered by using an automated temperature-water-based irrigation system and were fertilized twice a week with 2% Hakaphos (Compo Expert).

### Obtainment of pollen tube secretomes

**Fig. 1:**
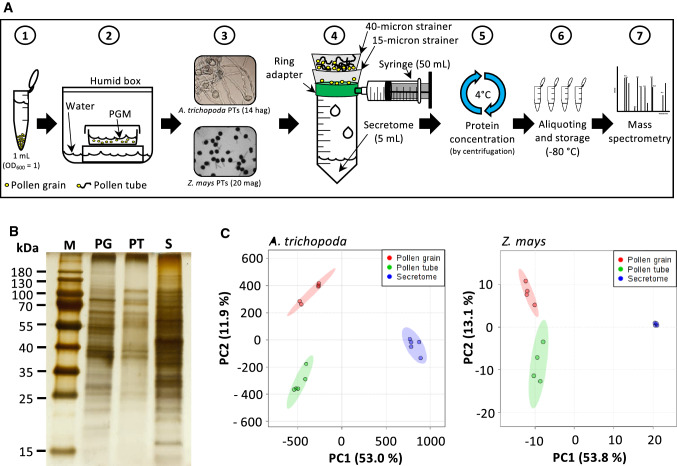
Procedure to obtain the pollen secretome from *Amborella trichopoda* and *Zea mays* and data quality analysis. **a** Schematic diagram describing the process of secretome obtainment in seven steps as indicated (see text for details). **b** Representative silver stained SDS-PAGEs showing differences in protein profiles of pollen grains (PG), pollen tubes (PT) and secretome (S) from *A. trichopoda*. **c** Principal component analysis (PCA) of the proteomic profile of *A. trichopoda* and *Z. mays*. Abbreviations: hag, hours after germination; mag, minutes after germination; PC, principal component; PGM, pollen germination medium

In order to obtain proteins secreted from growing pollen tubes (secretome), mature pollen grains from Amborella were isolated as described in Flores-Tornero et al. (2020). In brief, male flowers were rehydrated for 30 min and resuspended in fresh sterile-filtered Amborella pollen germination medium (5% sucrose, 300 μg/mL Ca(NO_3_)_2_, 200 μg/mL MgSO_4_, 100 μg/mL KNO_3_, 100 μg/mL H_3_BO_3_ and pH 5.0). After several filtering steps with pluriStrainer® 70–30 and 15-micron pore size strainers, clean pollen was obtained as a beige-white clean film on the 15-micron strainer and placed in a 1.5-mL collection tube. Fifty milligrams of clean pollen was immediately frozen in liquid nitrogen for protein extraction and stored at −80 ºC. The rest of the clean pollen was resuspended in pollen germination media, and 1 ml of pollen suspension (OD_600_ = 1) was diluted to a final volume of 5 mL, poured into 5.5-cm-diameter glass Petri dishes and incubated in a humid chamber at room temperature for up to 20 h on the laboratory bench. Fresh pollen was harvested directly from maize tassels in the morning, and 50 mg pollen was resuspended per 1 mL maize pollen germination medium (15% sucrose, 0.06% Ca(NO_3_)_2_, 0.02% MgSO_4_, 0.01% KNO_3_, pH 5.0) and incubated for 20 min at room temperature. After incubation, successful pollen germination and tube formation in Amborella and maize samples was confirmed with a Zeiss Axioscope and a Nikon Eclipse TE2000-S inverted microscope, respectively. The solution containing pollen tubes and its secreted proteins was filtered and pollen tubes were retained by filtration through a 40-micron strainer, whereas the non-germinated pollen grains were retained in the 15-micron strainer (see also Fig. 1a for an overview). Fifty milligrams of pollen tubes was collected from the surface of the 40-micron strainer with a spatula, placed in a 1.5-mL collection tube, immediately frozen in liquid nitrogen for protein extraction and stored at −80 ºC. The 5 mL flowthrough containing secreted proteins was concentrated at 4 ºC with Amicon® Ultra-centrifugal filter 3 kDa MWCO according to the manufacturer instructions, aliquoted into 25 µL volume and stored at −80 ºC. The total number of independent biological replicates used to extract proteins for pollen grains, pollen tubes and secretome was each 6 for Amborella and 4 for maize.

### Protein extraction and LC–MS/MS analysis

Total proteins from 50 mg of pollen grains and pollen tubes were extracted by grinding samples with liquid nitrogen in a mortar and subsequent addition of 250 μL of ice-cold extraction buffer (50 mM Tris/HCl, 150 mM NaCl, 0.1% sodium deoxycholate, 0.1% Triton-X100, 1 mM PMSF, pH 8.0). Pollen protein extracts were washed twice in a bench centrifuge (13,000 rpm, 10 min, 4 °C), distributed in aliquots of 50 μL and stored at −80 ºC. To analyze protein extracts, 10 μg of each biological replicate from pollen grains, pollen tubes and secreted proteins were loaded on a 12% SDS-PAGE using PageRuler™ Prestained Protein Ladder as marker (Thermo Fischer) and detected by silver staining according to Chevallet et al. (2006). Proteomic analyses were done as described (Hafidh et al. 2016). In short, protein solutions were processed by filter-aided sample preparation (FASP) using the Microcon device with MWCO 30 kDa (Merck Millipore) including alkylation (iodoacetamide, Sigma-Aldrich) and digestion step (trypsin, Promega). Liquid chromatography/mass spectrometric (LC–MS/MS) analyses of peptide mixtures were done using the UltiMate 3000 RSLCnano system connected to an Orbitrap Elite hybrid mass spectrometer (Thermo Fisher Scientific). MS data were acquired in a data-dependent strategy selecting up to top 10 precursors for higher-energy collisional dissociation (HCD) fragmentation. The analysis of mass spectrometric raw data files was carried out using the Proteome Discoverer software (Thermo Fisher Scientific; version 1.4) with in-house Mascot (Matrix Science; version 2.6) and Sequest search engines utilization. MS/MS ion searches were done against the modified cRAP database (based on https://www.thegpm.org/crap/) and UniProtKB protein database for both *Amborella trichopoda* (https://www.uniprot.org/proteomes/UP000017836; downloaded 18.4.2016, number of protein sequences 27,371) and *Zea mays* (ftp://ftp.uniprot.org/pub/databases/uniprot/current_release/knowledgebase/reference_proteomes/Eukaryota/UP000007305_4577; version 2017–07; 39,441 protein sequences). Percolator was used for post-processing of search results. Peptides with *q* value < 0.01, rank 1 and at least 6 amino acids long were considered only. Proteins abundance was assessed using protein area calculated by Proteome Discoverer software (Thermo Fisher Scientific; version 1.4). Peptides were either unambiguously assigned to a single protein record in UniProt database (Accession) or could match several proteins. In this case, the accessions of all these matching candidates were combined into supergroups (SG) and listed according to their match probability. The most probable candidate is identified as “master protein” and is always listed in the first position. Each SG was quantified as maximum of intensities of proteins in a protein group or as 90% of the minimal observed intensity in a sample. Then, log10 transformation was applied, and the data were linearly normalized to equalize the median intensities in all samples.

### Bioinformatic analyses

A statistical overrepresentation of gene ontology (GO) terms related to biological process was obtained from PANTHER version 15.0 (https://www.pantherdb.org). The in silico predictions for classical and non-classical secretory pathways were done using the default settings in SecretomeP v2.0, SignalP v5.0 and ProSite (https://prosite.expasy.org). Transcriptomic data from Amborella and maize were obtained from CoNekT online database (https://evorepro.sbs.ntu.edu.sg). The proteomic data from *Nicotiana tabacum* were obtained from Supplementary Table S4 from the publication Hafidh et al. (2016). We further processed the data and considered only proteins present in all replicates from the tobacco secreted samples (a total of 1375 proteins). As most of these proteins either did not have a valid ID to obtain information from UniProtKB or were obsolete in that database, a manual re-annotation based on the respective amino acidic sequences was performed. Re-annotation was made by bulk blast of amino acidic sequences against the UniProtKB database. By this procedure, only information from 798 tobacco proteins was recovered and used in this comparative study.

## Results

### Establishment of a simple and reproducible procedure to obtain the secretome of in vitro grown pollen tubes

To study the composition and nature of pollen tube secretions (hereafter named as secretome), we first isolated four replicates of fresh pollen from maize and six from Amborella as described (Flores-Tornero et al. 2020). Pollen grains from each replicate were separated for later protein extraction, and the rest were used for obtaining the secretome as described in Fig. 1a. In general, this procedure consisted of resuspending pollen grains in pollen germination medium and incubation in a humid box to induce germination. After 14 h for Amborella and 20 min for maize, secretions of growing pollen tubes were collected in liquid medium as shown in Fig. 1a. After checking the presence of pollen tubes, pollen germination media containing secreted proteins were filtered to separate pollen tubes and ungerminated pollen grains, respectively. Retained pollen tubes were recovered and used for protein extraction, whereas germination media containing the secretome were further concentrated by subsequent centrifugation, aliquoted and stored at −80 °C for LC–MS/MS analysis.

Silver-stained SDS-PAGE gels showed that protein extractions from pollen grains (PG), pollen tubes (PT) and the secretome (S) of Amborella look very different (Fig. 1b). In maize, PG and PT samples appeared more similar, while the S sample was also strongly different (Supplemental Fig. S1). A principal component analysis (PCA) of the data obtained by LC–MS/MS confirmed this observation and additionally showed that all biological replicates generated from the same sample group locate closely together showing the reproducibility of the data generated (Fig. 1c). Moreover, the finding that the secretome data points of both species were very homogenous and very distant from those of pollen grains and pollen tubes, which plot more closer to each other, further indicates that the secretome is not contaminated by burst pollen tubes.

### About 70% proteins detected in the pollen tube secretome are also in silico predicted to be secreted

**Fig. 2:**
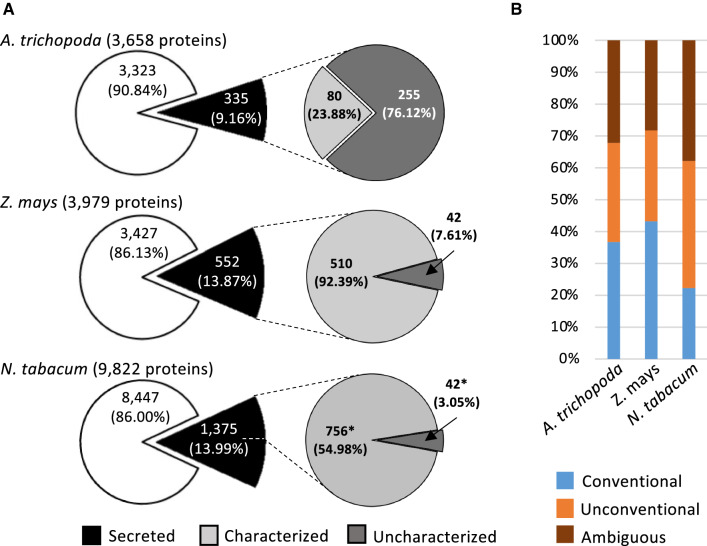
Statistical analysis of proteomic data obtained from pollen grains, pollen tubes and their secretome of *A. trichopoda, Z. mays* and *Nicotiana tabacum*. **a** Total number of proteins classified as “secreted” (detected in at least two biological replicates in the secretome data), “characterized” (described in the proteomic report) or “uncharacterized” (without description in the proteomic report). (*) Number of *N. tabacum* proteins with available UniProt ID (see text for details). **b** Predicted relative number of secreted proteins for conventional and unconventional secretory pathways, respectively

In order to analyze the composition of the secretomes generated and to compare it with proteins present in pollen grains and pollen tubes, respectively, samples from Amborella and maize were first analyzed by LC–MS/MS. Complete proteomic reports are provided in Supplemental Table S1 (Amborella) and Supplemental Table S2 (maize), respectively. Additionally, data for the tobacco secretome that has been generated in a previous study (Hafidh et al. 2016) were extracted from the corresponding supplementary material, further processed as described in Materials and Methods and is now provided as Supplemental Table S3 (tobacco). A total of 3658 proteins in all samples were detected in Amborella, 3979 in maize and 9822 in tobacco. For further analysis, we considered as “secreted” only those proteins with at least one peptide detected in at least two biological replicates of the secretome samples. About 12% of all proteins matched this criterion in all three plant species (Fig. 2a). Totally, 335 proteins were considered to be secreted from Amborella pollen tubes, 552 from maize and 1375 from tobacco pollen tubes. However, in contrast to Amborella and maize, about 42% proteins detected in the tobacco secretome did not have a valid ID and we thus continued only with the 798 proteins that were registered in UniProtKB and which were clearly associated with tobacco.

The majority of proteins detected in the Amborella samples (76%) were initially reported as “uncharacterized.” In maize and tobacco, these numbers were much lower (7.6% and 3%, respectively). In order to obtain more information about functions of detected proteins, we developed a strategy outlined in Supplemental Table S4a. In brief, we first searched in the current version of UniProtKB database and then in BLASTP and BLASTP for conserved protein domains. With this procedure, we could obtain already a description for many of these proteins. For those that remained uncharacterized, a BLASTP search was performed. In the case of maize and tobacco, a best-hit BLASTP search retrieved proteins exclusively described in other Poaceae or Solanaceae species, respectively (Supplemental Table S4c, d). In contrast, for Amborella the best-hit results were less specific, and they were found in many different plant species, ranging from other basal angiosperms to eudicots, monocots or even gymnosperms like *Picea sitchensis* (Supplemental Table S4b). Notably, at the end of this procedure there were only four uncharacterized proteins left in Amborella, 17 in maize and 10 in tobacco. These were described as “unknown” and labeled in yellow in Supplemental Table S4b, c, d.

Next, we investigated the proportion of proteins predicted to be secreted. We performed an in silico analysis to distinguish between the “conventional” and “unconventional” secretory pathway (see also Hafidh et al. 2016). As “conventional,” we classified proteins that contain a signal peptide for secretion via the ER and Golgi. Proteins were categorized as “unconventional” if they were predicted to be secreted but lack a typical signal peptide and as “ambiguous” those that were unclear. Unconventionally secreted proteins were predicted by using the SecretomeP 2.0 online server that uses algorithms that recognize the presence of common motifs and sequences that have been previously found in many proteins secreted by non-classical paths.

Notably, as shown in Fig. 2b, the Amborella secretome showed equal preference for conventional (36.7%) and unconventional secretion (31.1%), respectively, whereas in the maize secretome the conventional secretion was prevalent (43.2%; unconventional 28.6%). In tobacco, the unconventional pathway (39.9%) seemed to be expanded, while the conventional was reduced (22.3%). In conclusion, these numbers indicated that we detected proper secretomes from all three species and that many proteins are secreted by unconventional pathways. However, the significantly different numbers of detected proteins and annotation problems with data from tobacco do not allow yet conclusions about evolutionary aspects of prevalence of secretory pathways among different angiosperms.

### Secreted pollen tube proteins are especially enriched in functions associated with the cell wall, signaling and energy metabolism

**Fig. 3:**
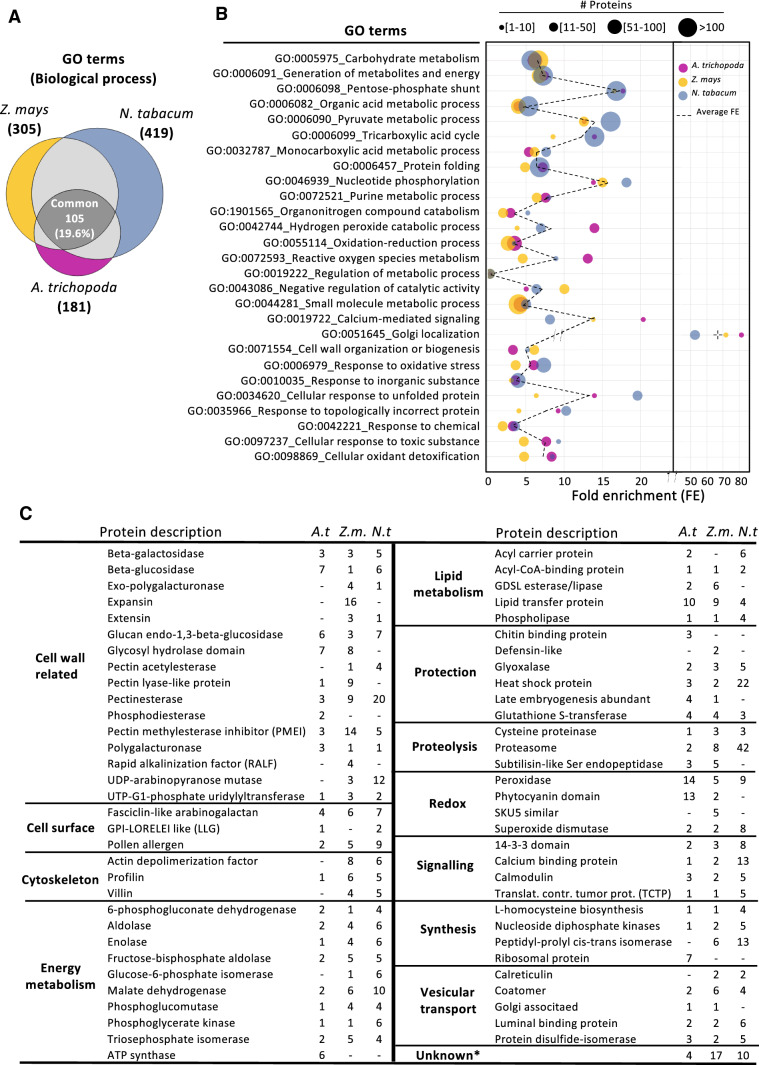
GO term analysis of secreted proteins from *A. trichopoda*, *Z. mays* and *N. tabacum*. **a** Venn diagram representing in brackets the total number of significantly enriched GO terms for biological processes in each species and the overlapping number. **b** Overlapping GO terms of all three species, number of detected proteins in each category, their fold enrichment (FE) and mean value indicated by a dashed line. **c** Most relevant secreted proteins when comparing *A. trichopoda* (*A.t.*), *Z. mays* (*Z.m*.) and *N. tabacum* (*N.t.*) pollen secretomes. (*) No available information (see Supplemental Table S3)

To gain insights into biological functions of secreted proteins, a gene ontology (GO) terms enrichment analysis was performed by using PANTHER v15.0 (Supplemental Table S5) using the above-mentioned 335 proteins from Amborella, 552 from maize and 798 from tobacco. As shown in Fig. 3a, a total of 181 significantly enriched GO terms were found for the Amborella secretome, 305 for maize and 419 for tobacco, with an overlap of 105 between the three species. This indicated that 37% GO terms overlap between the basal angiosperm Amborella and the other two species, but only 26% in the maize and 20% of the tobacco secretomes, indicating a higher specificity of functions in more recently diverged sister clade angiosperms. However, many proteins occur in more than one GO term and the number of different GO terms increases with the number of proteins; thus, these conclusions should be taken with care. The enrichment of the main common GO terms and the number of proteins that are involved in each of them are represented in Fig. 3b. This figure showed that enriched biological processes related to energy metabolism, signaling, Golgi localization or the cell wall are on average equally represented in the three species, whereas processes related to reactive oxygen species (ROS), calcium-mediated signaling or unfolded protein response are enriched either specifically in Amborella or tobacco.

For a deeper analysis, the description of all proteins in the secretome data was associated with a biological function and the presence compared for each category. As shown in Fig. 3c, proteins associated with the cell wall, cell surface and signaling including vesicular transport, but also energy and lipid metabolism as well as proteolysis were present in the secretomes. With the exception of proteins associated with redox processes as well as ATP synthase components and ribosomal proteins, there was no significant enrichment of proteins in the Amborella secretome compared with the other species. The latter two protein groups hint at a minor contamination in the Amborella samples from burst pollen tubes, which could be associated with the fact that pollen grains had to be incubated for 14 hs to generate tubes of a significant length. Compared with the other two species, there is a remarkable enrichment of expansins, pectin methylesterase inhibitors (PMEIs), pectin lyases and rapid alkalinization factors (RALFs) in maize. Cell wall enzymes like pectinesterases and UDP-arabinopyranose mutases are enriched in tobacco, but also heat shock proteins and proteasome components. The latter two categories indicate also for tobacco a slight contamination with cytosolic proteins. In summary, similar biological processes were enriched in the secretomes of the three selected angiosperm species. Proteins enriched in maize or tobacco, respectively, points toward differences on pollen tube cell wall composition and pollen tube signaling.

### Majority of most abundant secreted proteins are either cell wall-modifying enzymes or small cysteine-rich proteins (CRPs)

**Fig. 4:**
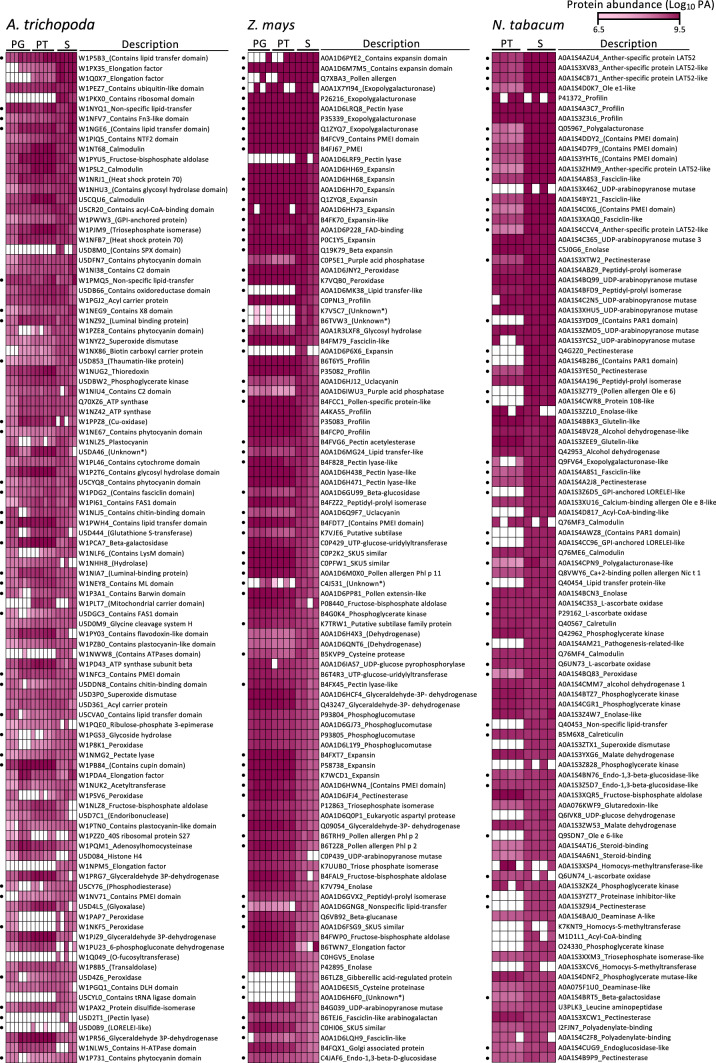
Identity of top 100 most abundant pollen tube secreted proteins from *A. trichopoda*, *Z. mays* and *N. tabacum*. Each cell represents the log10-transformed protein area of a given protein accession in parts per million (ppm). White cells represent the absence of protein. Proteins were sorted according to the highest number in the secretome data on top. Features from uncharacterized proteins are given in brackets. (●) Proteins with predicted signal peptide. *Proteins without any available information. *PG* pollen grain, *PT* pollen tube, *S* secretome

Next, we studied the identity of the top 100 most abundant proteins in the secretome samples, the presence of a signal peptide, and compared their abundance with that in pollen grain and pollen tube samples (Fig. 4). Protein abundance was calculated by the number of mapped peptides in each sample category for a given protein. Although this strategy discriminates against smaller proteins, we found that especially a number of smaller proteins including candidate signaling proteins were very abundant. Among the most often detected proteins in the Amborella samples are a larger number of different lipid-binding/transfer proteins (LTPs), elongation factors and a protein containing a ribosomal protein domain. As reported already above, especially different expansins are highly abundant in the secretome of maize, but also polygalacturonases, pectin lyases, peroxidases and small cysteine-rich proteins like LTPs, PMEI and pollen allergen Ole e1 confirming the data shown in Fig. 3c. Small cysteine-rich proteins representing also candidate signaling proteins like LAT52, pollen allergen Ole e1/6, PMEI and LTPs belong also to the most abundant proteins in the tobacco secretome. The second most abundant category is proteins involved in cell wall modification like polygalacturonases, UDP-arabinopyranose mutases and pectinesterases.

An in silico analysis revealed that 33%, 63% and 45% of the top 100 Amborella, maize and tobacco proteins contained a signal peptide. This number is similar to the number of all proteins in Amborella (see above), but significantly higher compared with the numbers for the conventional secretory pathway reported above for all maize and tobacco proteins, respectively. Notably, while most proteins were detected in all sample categories, proteins exclusively detected in the secretome of Amborella are above-mentioned protein containing a ribosomal protein domain, proteins with an SPX domain and tRNA ligase domain, respectively, as well as the cell wall enzymes O-fucosyltransferase and pectin lyase. The latter was also exclusively detected in the secretome of maize in addition to an expansin, a gibberellic acid-regulated protein, a cysteine proteinase and small unknown proteins. Abundant proteins present in the secretome, but not in the pollen tube samples of tobacco, included cell wall enzymes like UDP-arabinopyranose mutase and pectinesterase, but also small CRPs like pollen allergens and LTPs as well as two GPI-anchored LORELEI-like proteins, whose homologs were also found in the Amborella secretome.

**Fig. 5:**
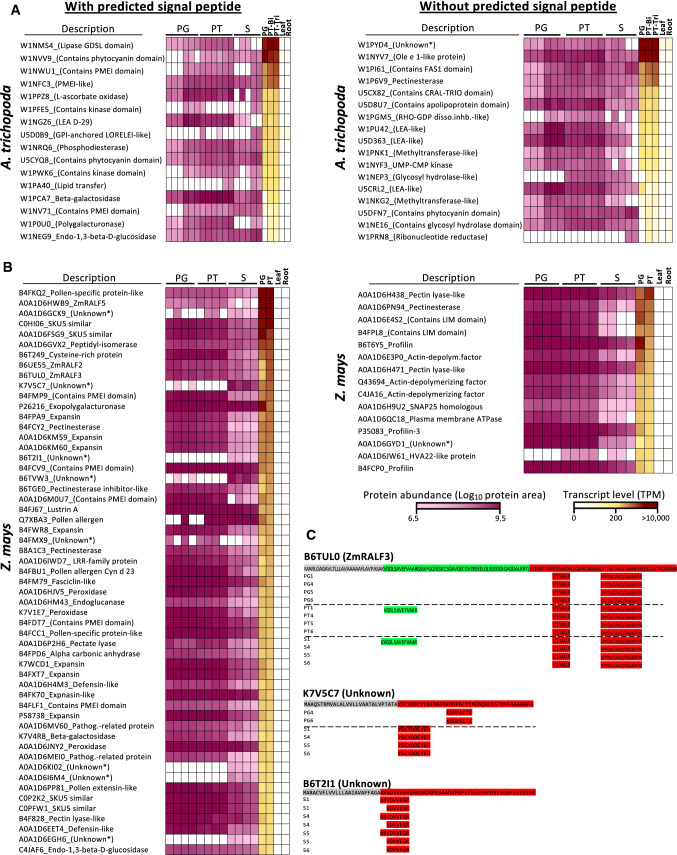
Comparison of proteomic and transcriptomic heatmaps of top expressed genes in pollen grains and pollen tubes of *A. trichopoda* and *Z. mays* as well as examples of mapped peptides. Genes and corresponding proteins were sorted with those showing the highest transcript levels on top. Two vegetative tissues are also included. **a** and **b** Proteins whose genes display at least 100 TPM expression levels in pollen tubes at the tricellular stage of *A. trichopoda* and 1000 TPM in pollen tubes of *Z. mays* are shown. Left blocks display proteins with predicted signal peptides and right blocks those lacking a predicted signal peptide. Color code for protein abundance and transcript level is indicated. Each biological protein sample is indicated. Average transcript levels were taken from Flores-Tornero et al. (2020). White cells represent the absence of protein or transcript. Features from uncharacterized proteins are given in brackets, and asterisks mark proteins without any available information. **c** Three examples showing mapping of identified peptides to small secreted proteins. *PG* pollen grain, *PT* pollen tube, *S* secretome

Finally, we wanted to explore the correlation between high transcript levels with abundancy of secreted proteins. Transcriptomic data for pollen grains and pollen tubes as well as vegetative control tissues were available in the CoNekT database (https://evorepro.sbs.ntu.edu.sg) for Amborella and maize. Similar data were not available for tobacco, and we thus restricted our final analyses on these two species. We selected all secreted proteins whose transcript levels were specifically expressed in male gametophytes with values of at least 100 transcripts per million (TPM) in pollen tubes of Amborella (Fig. 5a) and 1000 TPM in maize (Fig. 5b), respectively. Proteins were ordered in a manner that the strongest expressed genes were shown at the top. Additionally, we further classified proteins according to the presence or absence of a signal peptide. We observed that in Amborella there was an equal number of highly expressed genes encoding proteins with a signal peptide (16 proteins) and without (17 proteins). In maize, this number was approximately four times higher for genes encoding proteins with a signal peptide (55 proteins) compared with those lacking a signal peptide (15 proteins). This analysis detected the majority of the top 100 most abundant proteins (Fig. 4) indicating a strong correlation between transcriptomic and proteomic data. A few strongly expressed genes encoding, for example, a GPI-anchored LORELEI-like protein or LTPs in Amborella as well as a few unknown proteins in maize were only detected in few protein samples indicating low translation and high protein turnover rates, respectively. Notably, in the categories with a predicted signal peptide, almost all strongly expressed genes in both species encode either (i) CRPs like PMEIs, LTPs, RALFs, pollen allergens, defensin-likes and others or (ii) cell wall-modifying enzymes like galactosidases, glucanases, polygalacturonases, pectinesterases, pectate lyases, expansins and peroxidases as well as (iii) a few small proteins of unknown functions. The latter category is especially interesting as it includes with proteins A0A1D6GCK9, K7V5C7, B6T2I1, B6TVW3, A0A1D6KI02, A0A1D6EGH6 and A0A1D6I6M4 small novel secreted proteins that are described here for the first time and which are almost exclusively enriched in the secretome. As shown in Fig. 5c, K7V5C7, for example, encodes a predicted mature peptide of 42 amino acids likely stabilized by 6 cysteines, while B6T2I1 generates a predicted secreted peptide of 48 amino acids that might be further processed as it lacks cysteines and we only detected peptides of 7 and 9 amino acids length, respectively, after the predicted *N*-terminal cleavage site. For other secreted proteins like RALF3 (Fig. 5c), we detected pro-protein peptides in the secretome, indicating that processing to fully mature proteins occurs (also) in the apoplast. In summary, the majority of most abundant secreted proteins or most strongly expressed genes encoding secreted proteins are either cell wall-modifying enzymes or CRPs. Finally, we detected novel secreted proteins and showed how these data can be used to study also processing of secreted proteins.

## Discussion

### Enrichment of cell wall enzymes and CRPs in pollen tube secretomes

The observation that we detected especially cell wall enzymes and CRPs in pollen tube secretomes was not surprising taking into consideration that these belong also to the strongly expressed genes (e.g., Bokvaj et al. 2015; Conze et al. 2017; Tan et al. 2018). Moreover, pollen tubes are fast growing plant cells that require a solid cell wall and that communicate intensively with the surrounding maternal tissues. Surprisingly, we also found many enzymes involved in lipid and energy metabolism, but also in proteolysis and redox processes as well as predicted cytoplasmic proteins like cytoskeletal components and heat shock proteins. These observations point toward the possibility that the secretomes might have been contaminated with cytoplasmic proteins from burst pollen tubes. However, > 70% identified proteins were predicted to be secreted; the composition of the secretomes was highly different compared with that from pollen tubes in each species and very homogenous in itself in independent experiments as shown by PCA analyses. Moreover, gene ontology terms associated with the above processes were significantly overrepresented in the secretomes of all three species compared with other tissues, indicating that contaminations were negligible.

During their journey, pollen tubes require many nutrients from the style, a lot of energy, but also structural components to generate cell wall material and membranes. Thus, it is not unlikely that many enzymes and structural components were secreted to contribute to the high speed of pollen tube growth. Notably, a similar observation has also been reported recently in studies about reproductive secretions in gymnosperms (Prior et al. 2019): Predicted intracellular and cytoplasmic proteins were detected in pollination droplets of naked ovules. The biological relevance of these proteins in the extracellular space is not clear. Moreover, another study about pollen tube secretions in *Olea europaea* not only described the presence of GAPDH, fructokinases and actin in the secretome, but also pointed toward the relevance of the unconventional secretory pathway to secrete cytosolic proteins (Prado et al. 2014). To which extent the in vitro obtained secretome data reflect the in vivo secretomes at different stages of the pollen tube journey is also unclear. It has been shown that germination media influence the pollen tube transcriptome in *Arabidopsis thaliana* (da Costa et al. 2013) and thus may also have an impact on the secretome. Moreover, in the same species it was shown that the pollen tube transcriptome also changes during growth through the stigma and style (Qin et al. 2009) and thus likely also leads to changes in the secretome composition. So far, differences in the secretome from pollen tubes germinated in vitro and semi-in vivo have only been reported in tobacco (Hafidh et al. 2016) and were discussed to occur due to the intensive cross talk between pollen tubes and female tissues during stigma–style penetration and growth (Dresselhaus and Franklin-Tong 2013) that is lacking during pollen tube growth in vitro.

### Correlation of secreted proteins with pollen tube growth rate and defense responses

Notably, compared with Amborella, significantly more enzymes required for cell wall synthesis and modification as well as energy metabolism were detected in the maize and tobacco secretomes, explaining that their pollen tubes are capable to grow much faster. Additionally, this may also reflect the length of the journey: While pollen tubes in maize travel up to 30 cm (Zhou et al. 2017), tubes of tobacco grow around 4.5 cm (Cheung et al. 2000) and those of Amborella only about 0.12 cm (Williams 2009). These findings may also reflect differences in cell wall compositions, thickness and elasticity among the three species. For instance, the presence of many expansins in maize involved in cell wall loosening likely reflects the necessity of elastic walls during high-speed tube growth as cell walls of grasses also contain low amounts of pectins and xyloglucans compared with other angiosperms (Cosgrove 2015). The low number of beta-glucosidases detected in the maize secretome correlates with the low number of genes encoding for beta-glucosidases in this species compared to others (Gomez-Anduro et al. 2011). PMEIs that regulate cell wall elasticity (Wormit and Usadel 2018) are abundantly transcribed and translated in all three species investigated. However, 35 of 49 PMEIs were reported to be specifically transcribed and translated in pollen grains of maize (Zhang et al. 2019). Altogether, this suggests that the wall of fast-growing maize pollen tubes remains very elastic during growth, but hints also to the possibility that cell walls of maternal tissues have to be loosened at high speed to allow pollen tube penetration. Tobacco secretes abundantly UDP-arabinopyranose mutase, which correlates with findings in other tissues, showing that arabinoxyloglucans are especially abundant in cell walls of solanaceous plants (Honta et al. 2018).

In addition to proteins involved in energy and lipid metabolism as well as cell wall synthesis and modification, many CRPs were detected in the secretomes. Allergens like Pollen Ole e 1 have been abundantly detected in maize and tobacco. They have also been reported previously in in vitro pollen tube secretions from *Olea europaea* and were suggested to play a possible role in signaling (Alché et al. 2004). LTPs, which are considered as key proteins in lipid barrier polymer synthesis and extracellular signaling in many tissues (Salminen et al. 2016), were also shown as components regulating pollen tube adhesion to the stigma and style in *Lilium longiflorum* (Park et al. 2000) and were detected in all three species. Genes encoding RALFs that have been shown to regulate pollen tube growth and cell wall integrity are massively and specifically transcribed in both male and female gametophytes of all angiosperm clades (Campbell and Turner 2017; Flores-Tornero et al. 2019, 2020; Ge et al. 2017; Mecchia et al. 2017). Although we detected RALFs in the maize secretome, they were not among the top 100 most abundantly secreted proteins and some were only found in the pollen grain and pollen tube fractions, respectively. Similarly in Amborella, where members of the RALF family belong to the highly expressed genes in male gametophytes (Flores-Tornero et al. 2020), proteins were detected only in pollen grain and pollen tube protein extractions, respectively. In *Arabidopsis thaliana*, RALF4/19 have been characterized as small peptides that are highly transcribed and abundantly secreted from pollen tubes (Ge et al. 2017; Mecchia et al. 2017). It could thus be expected that their homologs in the eudicot tobacco are also abundant in the secretome, which is not the case. This finding points toward the possibility that RALF peptides are less stable in the extracellular space compared with LTPs, Pollen Ole e 1 s and other larger CRPs. The presence of cysteine proteinases, subtilisin-like serine endopeptidase and other aminopeptidases in pollen tube secretomes supports the hypothesis that some proteins are rapidly degraded, while others are more stable.

Proteins related to protection like chitin binding proteins, thaumatins, LEA proteins or proteins containing Barwin or ML domains are among the top 100 most abundant proteins in the Amborella, but not in the maize and tobacco secretomes, respectively. Notably, their homologs were also present in pollination drops from gymnosperms (Prior et al. 2019). This observation hints toward the hypothesis that wild plant species spend more efforts and energy into defense and thus contain higher amounts of these proteins in their apoplasts, while cultivated species like maize and tobacco are bred for high yield in sacrifice for the generation of protection proteins.

## Conclusions and outlook

In conclusion, we provide here a simple and very robust procedure that could potentially be used for many other angiosperms to obtain pollen tube secretomes. The analysis of the secretome from three phylogenetically distant angiosperm clades shows that—similar to other plant tissues (Krause et al. 2013)—on average both, conventional and unconventional secretory pathways are used at similar rates to secrete proteins from growing pollen tubes to the apoplast. Identified cell wall enzymes differ in abundance significantly among the three plant species analyzed likely reflecting differences in cell wall types and their elasticity in response to varying pollen tube growth speed and length. Novel small secreted proteins or peptides were discovered in secretomes and partially unprocessed pro-peptides pointing toward final cleavage steps in the apoplast, which is rich in proteases. For future studies, we now recommend to extract higher protein amounts and generate more mapped peptides after LC MS/MS analysis as the data generated are very useful to elucidate also the ultimate sequence and posttranslational modification(s) of secreted mature proteins. Improvements in LC MS/MS technology may detect more and less abundant proteins in pollen tube secretomes in future studies, but we think that the data presented provide already a valuable resource for gene/protein discovery for functional studies as well as for comparative studies involving further angiosperm species to better understand the evolution, function and specificity of pollen tubes and their journey.

### Author contributions statement

FV initiated the study. SS and TD designed the study. MFT and LW generated the samples, SH and DH performed the analyses, DP and ZZ performed the proteomic analyses, and MFT analyzed the data and wrote the manuscript with TD.

## Electronic supplementary material

Below is the link to the electronic supplementary material.

Supplementary file1 (DOCX 347 kb)

Supplementary file2 (XLSX 23557 kb)

## References

[CR1] Agrawal GK, Jwa NS, Lebrun MH, Job D, Rakwal R (2010). Plant secretome: unlocking secrets of the secreted proteins. Proteomics.

[CR2] Alché JdD, M’rani-Alaoui M, Castro AJ, Rodríguez-García MI (2004). Ole e 1, the major allergen from olive (*Olea europaea* L.) pollen, increases its expression and is released to the culture medium during *in vitro* germination. Plant Cell Physiol.

[CR3] Bircheneder S, Dresselhaus T (2016). Why cellular communication during plant reproduction is particularly mediated by CRP signalling. J Exp Bot.

[CR4] Bokvaj P, Hafidh S, Honys D (2015). Transcriptome profiling of male gametophyte development in *Nicotiana tabacum*. Genom Data.

[CR5] Campbell L, Turner SR (2017). A comprehensive analysis of RALF proteins in green plants suggests there are two distinct functional groups. Front Plant Sci.

[CR6] Chae K, Lord EM (2011). Pollen tube growth and guidance: roles of small secreted proteins. Annals Bot.

[CR7] Chae K, Gonong BJ, Kim S-C, Kieslich CA, Morikis D, Balasubramanian S, Lord EM (2010). A multifaceted study of stigma/style cysteine-rich adhesin (SCA)-like Arabidopsis lipid transfer proteins (LTPs) suggests diversified roles for these LTPs in plant growth and reproduction. J Exp Bot.

[CR8] Cheung AY (2000). Pollen–pistil interactions in *Nicotiana tabacum*. Annals Bot.

[CR9] Chevallet M, Luche S, Rabilloud T (2006). Silver staining of proteins in polyacrylamide gels. Nat Protoc.

[CR10] Conze LL, Berlin S, Le Bail A, Kost B (2017). Transcriptome profiling of tobacco (*Nicotiana tabacum*) pollen and pollen tubes. BMC Genomics.

[CR11] Cosgrove DJ (2015). Plant expansins: diversity and interactions with plant cell walls. Curr Opin Plant Biol.

[CR12] da Costa ML, Pereira LG, Coimbra S (2013). Growth media induces variation in cell wall associated gene expression in *Arabidopsis thaliana* pollen tube. Plants.

[CR13] Dehors J, Mareck A, Kiefer-Meyer MC, Menu-Bouaouiche L, Lehner A, Mollet JC (2019). Evolution of cell wall polymers in tip-growing land plant gametophytes: composition, distribution, functional aspects and their remodeling. Front Plant Sci.

[CR14] Dresselhaus T, Franklin-Tong N (2013). Male-female crosstalk during pollen germination, tube growth and guidance, and double fertilization. Mol Plant.

[CR15] Dresselhaus T, Sprunck S, Wessel GM (2016). Fertilization mechanisms in flowering plants. Curr Biol.

[CR16] Flores-Tornero M, Proost S, Mutwil M, Scutt CP, Dresselhaus T, Sprunck S (2019). Transcriptomics of manually isolated *Amborella trichopoda* egg apparatus cells. Plant Reprod.

[CR17] Flores-Tornero M (2020). Transcriptomic and proteomic insights into *Amborella trichopoda* male gametophyte functions. Plant Physiol.

[CR18] Ge Z (2017). Arabidopsis pollen tube integrity and sperm release are regulated by RALF-mediated signaling. Science.

[CR19] Gomez-Anduro G, Ceniceros-Ojeda EA, Casados-Vazquez LE, Bencivenni C, Sierra-Beltran A, Murillo-Amador B, Tiessen A (2011). Genome-wide analysis of the beta-glucosidase gene family in maize (*Zea mays* L. var B73). Plant Mol Biol.

[CR20] Hafidh S, Potesil D, Fila J, Capkova V, Zdrahal Z, Honys D (2016). Quantitative proteomics of the tobacco pollen tube secretome identifies novel pollen tube guidance proteins important for fertilization. Genome Biol.

[CR21] Honta H, Inamura T, Konishi T, Satoh S, Iwai H (2018). UDP-arabinopyranose mutase gene expressions are required for the biosynthesis of the arabinose side chain of both pectin and arabinoxyloglucan, and normal leaf expansion in *Nicotiana tabacum*. J Plant Res.

[CR22] Huang Q, Dresselhaus T, Gu H, Qu LJ (2015). Active role of small peptides in arabidopsis reproduction: expression evidence. J Integr Plant Biol.

[CR23] Johnson MA, Harper JF, Palanivelu R (2019). A fruitful journey: pollen tube navigation from germination to fertilization. Annu Rev Plant Biol.

[CR24] Krause C, Richter S, Knoll C, Jürgens G (2013). Plant secretome - from cellular process to biological activity. Biochimica Biophysica Acta.

[CR25] Maruyama D, Higashiyama T (2016). The end of temptation: the elimination of persistent synergid cell identity. Curr Opin Plant Biol.

[CR26] Mecchia MA (2017). RALF4/19 peptides interact with LRX proteins to control pollen tube growth in Arabidopsis. Science.

[CR27] Mollet JC, Leroux C, Dardelle F, Lehner A (2013). Cell wall composition, biosynthesis and remodeling during pollen tube growth. Plants.

[CR28] Muschietti J, Dircks L, Vancanneyt G, McCormick S (1994). LAT52 protein is essential for tomato pollen development: pollen expressing antisense LAT52 RNA hydrates and germinates abnormally and cannot achieve fertilization. Plant J.

[CR29] Park SY, Jauh GY, Mollet JC, Eckard KJ, Nothnagel EA, Walling LL, Lord EM (2000). A lipid transfer-like protein is necessary for lily pollen tube adhesion to an in vitro stylar matrix. Plant Cell.

[CR30] Prado N, Alche Jde D, Casado-Vela J, Mas S, Villalba M, Rodriguez R, Batanero E (2014). Nanovesicles are secreted during pollen germination and pollen tube growth: a possible role in fertilization. Mol Plant.

[CR31] Prior N (2019). Complex reproductive secretions occur in all extant gymnosperm lineages: a proteomic survey of gymnosperm pollination drops. Plant Reprod.

[CR32] Qin Y (2009). Penetration of the stigma and style elicits a novel transcriptome in pollen tubes, pointing to genes critical for growth in a pistil. PLoS Genet.

[CR33] Qu LJ, Li L, Lan Z, Dresselhaus T (2015). Peptide signalling during the pollen tube journey and double fertilization. J Exp Bot.

[CR34] Salminen TA, Blomqvist K, Edqvist J (2016). Lipid transfer proteins: classification, nomenclature, structure, and function. Planta.

[CR35] Tan H, Zhang J, Qi X, Ye W, Wang X, Xiang X (2018). Integrated metabolite profiling and transcriptome analysis reveals a dynamic metabolic exchange between pollen tubes and the style during fertilization of *Brassica napus*. Plant Mol Biol.

[CR36] Williams JH (2009). *Amborella trichopoda* (Amborellaceae) and the evolutionary developmental origins of the angiosperm progamic phase. Am J Bot.

[CR37] Wormit A, Usadel B (2018). The multifaceted role of pectin methylesterase inhibitors (PMEIs). Intern J Mol Sci.

[CR38] Zhang J (2017). Sperm cells are passive cargo of the pollen tube in plant fertilization. Nat Plants.

[CR39] Zhang P, Wang H, Qin X, Chen K, Zhao J, Zhao Y, Yue B (2019). Genome-wide identification, phylogeny and expression analysis of the PME and PMEI gene families in maize. Sci Rep.

[CR40] Zhou LZ, Dresselhaus T (2019). Friend or foe: signaling mechanisms during double fertilization in flowering seed plants. Curr Top Dev Biol.

[CR41] Zhou LZ, Juranic M, Dresselhaus T (2017). Germline development and fertilization mechanisms in maize. Mol Plant.

