# Supplementary material for: Comparative analyses of angiosperm secretomes identify apoplastic pollen tube functions and novel secreted peptides
Source: Plant Reprod. 2020 Nov 30;34(1):47–60. doi: 10.1007/s00497-020-00399-5 (PMC7902602; doi:10.1007/s00497-020-00399-5)
Supplement: Supplementary file 1 — Supplementary file1 (DOCX 347 kb) [file 497_2020_399_MOESM1_ESM.docx]

**Supplementary material**

**
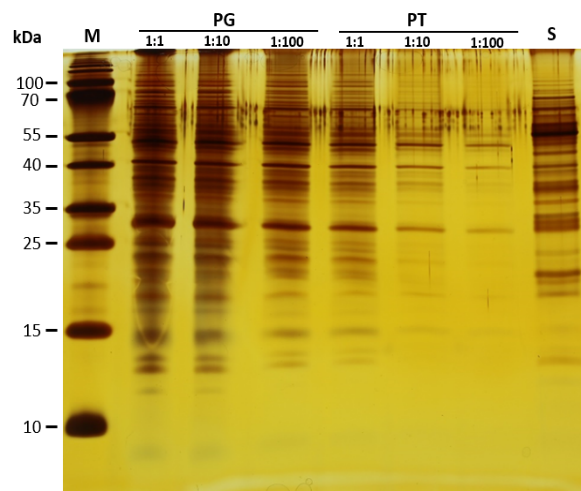
**

**Fig. S1** Silver stained SDS-PAGE showing dilutions of protein profiles of pollen grains (PG), pollen tubes (PT) and secretome (S) from *Z. mays*. M, marker. While pollen grain and pollen tube protein pattern are quite similar, the secretome appears very different.
